# Understanding the trends in international agreements on pricing and reimbursement for newly marketed medicines and their implications for access to medicines: a computational text analysis

**DOI:** 10.1186/s12992-020-00633-9

**Published:** 2020-10-14

**Authors:** Kyung-Bok Son

**Affiliations:** College of Pharmacy, Ewha Womans University, 52Ewhayeodae-gil, Seodaemun-gu, Seoul, 03760 South Korea

**Keywords:** Access to medicines, International agreements, Pricing and reimbursement, Procedural fairness

## Abstract

**Background:**

Health systems are struggling with unprecedented drug spending and governments have devised various policy options to manage high-priced medicines. Meanwhile, some pricing and reimbursement processes are currently moving under the jurisdiction of international agreements. This study aims to understand trends in international agreements from the perspectives of pricing and reimbursement policies for newly marketed medicines.

**Methods:**

We proposed the framework to interpret the international agreements as code and applied computational text analysis to understand international agreements as data. In particular, we selected the AUSFTA, KORUS, and TPP to assess the progress and evolution in international agreements and investigate the existing relevant content on the pricing and reimbursement of newly marketed medicines.

**Results:**

Similar to the provisions for intellectual property, the scope of international agreements regarding pricing and reimbursement decisions are broadened and strengthened. Over time, the domain of transparency, re-naming procedural fairness, has changed significantly more than the remaining domains. Pharmaceutical companies will have more opportunities to advocate for their positions, to protect their interests in decision processes, to investigate the decisions on listings and setting the amounts of reimbursement, and to challenge these decisions.

**Conclusions:**

Recently signed international agreements favor companies over governments with underscoring procedural fairness and timely access. However, access to affordable medicines is the goal towards which international agreements should aim. In a similar vein, substantial fairness and the accountability of companies should be discussed when negotiating agreements or adopting international agreements through domestic legislation.

## Background

Health systems are struggling with unprecedented drug spending with the marketing of high-priced medicines and sudden price increases among generic drugs [1–3]. Increased uncertainty about newly marketed medicines from the perspectives of clinical outcomes, cost-effectiveness, and budget impact and lack of transparency in decision-making processes are common challenges in the global medicine environment. Governments have devised various policy options to reduce the uncertainty in budget impacts and to manage pharmaceutical expenditure incurred by high-priced medicines [4–9]. Essential ways to address these challenges are pricing and reimbursement policies.

New trade and industry norms are expanding their scopes into low- and middle-income countries through international trade agreements [10, 11], implying that some pricing and reimbursement processes are currently moving under the jurisdiction of international agreements. Specifically, clauses on transparency and innovation have been included in various trade agreements [12–15]. The Korea-US Free Trade Agreement (KORUS) includes articles entitled “access to innovation” and “transparency”, and the Trans-Pacific Partnership (TPP) provides articles entitled “procedural fairness” and “transparency”. These articles could affect governments’ decisions on the pricing and reimbursement of newly marketed medicines [11].

Many researchers have been interested in international agreements and their impacts on access to medicines [16–21]. However, there are limitations in these studies. Their main interests have centered on intellectual property, including the protection of patents and undisclosed tests against unauthorized disclosure of clinical trial data. Scholars have analysed the trends in international agreements on pharmaceutical affairs with a focus on intellectual property, including patent linkage systems and data exclusivity [11, 22, 23]. In a similar vein, post-TRIPS intellectual property rights have been analyzed, and provisions potentially affecting domestic health and social policy settings have been suggested [24].

Little of the literature has discussed the impact of international agreements on pricing and reimbursement processes within member countries. Researchers have meaningfully analyzed the TPP and the Comprehensive and Progressive Agreement for Trans-Pacific Partnership (CPTPP) and suggested their implications on access to medicines from the perspectives of pricing and reimbursement policies [25–29]. However, the literature has been interested in the *“overall”* characteristics of *“the TPP or the CPTPP”*. In other words, the literature could not address trends in international agreements and their specific impact on pricing and reimbursement policies within the member countries. This study aims to understand trends in international agreements from the perspectives of pricing and reimbursement policies for newly marketed medicines. To this end, we proposed the framework to interpret international agreements as code and applied computational text analysis to understand international agreements as data.

### The rationale for a computational text analysis

Text has become an important data source in social science [30]. With the digitalization of texts and advances in data processing technology, computational text analysis has boomed over the past decades to explore research questions or to understand the ambiguous goals of persons or society [31, 32]. In particular, understanding international agreements is coming to involve big data rather than a small amounts of text [30, 33]. Traditionally, interpreting a treaty or extracting implications from complex judicial documents has required scholars’ in-depth intellectual engagement with limited number of texts, and scholars have made descriptive or normative claims. This approach has served as the basis of understanding international agreements for centuries. However, it has limitations in terms of limiting the amount of data that can be processed and the types of legal analysis that can be applied [30].

Few studies have embarked on transforming legal texts into data that can be the subject of computational text analysis. These studies adopting the computational approach put big data at the disposal of scholars and enable scholars to investigate international agreements in unprecedented depth and breadth [34–36]. Scholars in political economy have focused on the evolution of international investment agreements and evaluated their trends quantitatively through content analysis [34]. Particularly, they utilize a list of keywords distributed throughout the agreements to identify whether a specific content is present in the agreement and measure legal precision to represent the highest possible degree of precision [34]. Scholars in international law have proposed a novel approach to investigate great numbers of international agreements and measured textual similarities across the agreements [35]. Scholars in international studies have identified a sentiment dictionary used in the political communication literature and conducted text analysis of the rulings of the World Trade Organization [36].

## Methods

Recently, the scope of intellectual property in international agreements has been notably broadened and strengthened through bilateral and regional free trade agreements [37]. We selected the Australia-United States Free Trade Agreement (AUSFTA), KORUS, and TPP to assess the progress and evolution in international agreements and investigate the existing relevant content on the pricing and reimbursement of newly marketed medicines. Considering the decade-long gaps between the agreements, these three international agreements are suitable for noting the trends in international trade agreements.

The AUSFTA, a bilateral trade agreement between Australia and the United States, was signed in February 2004 and implemented in January 2005 [34]. Annex 2-C Pharmaceuticals and Side letters PBS, which are under Chapter 2, entitled “National Treatment and Market Access for Goods”, provides information about the pricing of and reimbursement process for medicines. KORUS is also a bilateral agreement; it was signed in 2007 but not ratified until 2012 [35, 36]. Chapter 5, entitled “Pharmaceutical Products and Medical Devices”, is closely related to the pricing and reimbursement process. Finally, the TPP is a plurilateral agreement among 12 countries, including the United States, Japan, and Australia [37]. The United States withdrew from the agreement in January 2017. However, the remaining 11 countries decided to proceed despite the absence of the United States and concluded negotiations in January 2018, renaming the treaty CPTPP. The CPTPP is a short-form treaty that incorporates by reference all the provisions of the TPP except those explicitly identified for suspension. Chapter 26, entitled “Transparency and Anti-Corruption”, and its Annex on Transparency and Procedural Fairness for Pharmaceutical Products and Medical Devices (Annex 26A) are relevant provisions. Note that Article 3 on Procedural Fairness in Annex 26A was suspended in the CPTPP. However, it is reasonable to assume that the suspended provision would reappear in international agreements as an important trade agenda. Thus, we included the TPP rather than the CPTPP as the subjects in our analysis.

We formulated the framework to understand international agreements from the perspective of the pricing and reimbursement process for newly marketed medicines. In particular, we undertook a purposive review of the literature on the impacts of international agreements on the pricing and reimbursement schemes in member countries [25–29] and the pricing and reimbursement process of newly marketed medicines in various countries [38–42]. Based on the framework, we applied two distinct approaches to understand international agreements: the law-as-code approach and the law-as-data approach. The first attempts to describe international agreements as a set of formal rules, while the latter uses computational techniques to extract quantitative information from texts in international agreements [43, 44]. In the law-as-code approach, we read and interpreted texts in eligible clauses, and then made descriptive and normative claims about the agreements, which is the core research methodology of law. In the law-as-data approach, we used natural language processing, which automatically processes full text, to transform international agreements into a numerical representation. In particular, we imported the text of the eligible provisions in the agreements, subdivided the text into words, and analyzed the words, including word frequencies and correlations among the subjects. Data management and analysis were performed using R statistical software (version 3.4.1). Particularly, the “Tidyverse” package in R statistical software was used to count word frequencies and to conduct a correlation test. Statistical significance was defined as a *p*-value less than 0.05.

## Results

### Suggested framework to understand international agreements on the pricing and reimbursement of pharmaceuticals

The pricing and reimbursement of pharmaceuticals has been a source of continuous policy debate [38–41]. Governments are concerned with health care expenditure and view pharmaceutical expenditure as a priority policy area [42]. Economic evaluation has a central role in drug pricing and reimbursement decisions by providing a method to establish the additional value of a new drug to society [40]. However, it is noteworthy that economic evaluation helps the governments assess the value of a newly marketed drug but not determine its price. The price of a drug can be set when pricing rules are adopted, implying that pricing rules are needed to specify the proportion of the added value to society [41]. Recently, new trade norms have expanded their scopes through international trade agreements, and some pricing and reimbursement policies are moving under the jurisdiction of international agreements. Few studies have specified the areas and their pathways that threaten access to medicines: intellectual property, investment, and transparency (or procedural requirements) [25–29].

Building upon the discussion of the pricing and reimbursement of pharmaceuticals and the impacts of international agreements on the pricing and reimbursement process, we categorized the framework into principles, process, and outcomes. Principles indicate the guiding values expected for pricing and reimbursement decisions in international agreements. The process indicates how decisions are made and/or the mechanisms through which policies are involved. Outcomes refer to the outputs of coordination between principles and process. Then, we reviewed the related provisions in international agreements to modify the frameworks. The KORUS states 8 provisions in Chapter 5: general provisions, access to innovation, transparency, dissemination of information, ethical business practices, regulatory cooperation, medicines and medical devices committee, and definitions. Similarly, the AUSFTA states 6 provisions in Annex 2-C: agreed principles, transparency, medicines working group, regulatory cooperation, dissemination of information, and definitions. At this stage, we found that the domains of international agreements were consistent with the proposed principles, process, and outcomes framework. Finally, we calibrated the framework into principles, transparency, and value for money.

### Interpreting international agreements as code

Table 1 presents the characteristics of the subjects and their eligible provisions corresponding to the framework. The scope of the agreements was expanded from pharmaceuticals to medical devices, and the content of the agreement was clarified in more detailed form particularly for transparency and procedural fairness. First, the KORUS and TPP include not only pharmaceuticals but also medical devices in the agreements, while the AUSFTA covers only pharmaceuticals. Furthermore, the TPP, as demonstrated in the headings of the chapter and its annex, clarifies that anticorruption and procedural fairness are under the scope of the agreement. Second, the AUSFTA presents the corresponding provisions in the annex and side letters with 6 provisions in 580 words and 4 provisions in 251 words, respectively. However, the KORUS and TPP articulate the provisions in the relevant chapter with 8 provisions in 1731 words and 17 provisions in 5208 words, respectively.

**Table 1:** The overall scopes of the subjects and the provisions corresponding to the framework

	AUSFTA		KORUS	TPP	
Signed / Implemented year	**2004 / 2005**		**2007 / 2012**	**2018 / 2018**	
Subjects	Annex 2-C - Pharmaceuticals	Side letters PBS	Chapter 5 – Pharmaceutical products and medical devices	Chapter 26 – Transparency and Anticorruption	Annex 26-A Transparency and Procedural Fairness for Pharmaceutical Products and Medical Devices
Composition	6 provisions and 580 words	4 provisions and 251 words	8 provisions and 1731 words	3 sections (definitions, transparency, anti-corruption), 12 provisions, 3136 words	5 provisions and 2072 words
Principles	1: Agreed Principles		1: General provisions	–	2: Principles
Value for money (innovation)	–		2: Access to innovation	–	–
Transparency	2: Transparency	4 provisions are related with transparency	3: Transparency	2: Publication 3: Administrative Proceedings 4: Review and Appeal 5: Provision of Information	3: Procedural Fairness
Others	3: Medicines Working Group 4: Regulatory Cooperation 5: Dissemination of Information 6: Definitions		4: Dissemination of Information 5: Ethical Business Practices 6: Regulatory Cooperation 7: Medicines and medical devices committee 8: Definitions	1: Definitions	1: Definitions 4: Dissemination of Information to Health Professionals and Consumers 5: Consultation

### Principles

Table 2 presents principles of the AUSFTA, KORUS, and TPP. The domains of principles are composed of aims and areas. Aims provide the purposes of the agreements. All the agreements clarify that their aims are to facilitate high-quality health care and to improve the health of their nationals (or public health).

**Table 2:** Comparison of principles provisions in AUSFTA, KORUS, and TPP

	AUSFTA	KORUS	TPP
Aims	Facilitating high quality health care and continued improvements in public health	Promoting the development of and facilitating access to high-quality patented and generic pharmaceutical products and medical devices, as a means of continuing to improve the health of their nationals.	Facilitating high-quality health care and continued improvements in public health for their nationals, including patients and the public.
Areas			
Public health	(a) the important role played by innovative pharmaceutical products in delivering high quality health care;	(a) adequate access to pharmaceutical products and medical devices in providing high quality health care; (b) patented and generic pharmaceutical products and medical devices in reducing other more costly medical expenditures;	(a) the importance of protecting and promoting public health and the important role played by pharmaceutical products and medical devices in delivering high-quality health care;
Research & development	(b) the importance of research and development in the pharmaceutical industry and of appropriate government support, including through intellectual property protection and other policies;	(d) appropriate government support of research and development in academic and commercial laboratories, intellectual property protections, and other incentives for innovation in the research and development of pharmaceutical products and medical devices;	(b) the importance of research and development, including innovation associated with research and development, related to pharmaceutical products and medical devices;
Access	(c) the need to promote timely and affordable access to innovative pharmaceuticals through transparent, expeditious, and accountable procedures	(e) promoting innovation and timely and affordable access to safe and effective pharmaceutical products and medical devices through transparent and accountable procedures	(c) the need to promote timely and affordable access to pharmaceutical products and medical devices, through transparent, impartial, expeditious and accountable procedures
Value	(d) the need to recognize the value of innovative pharmaceuticals through the operation of competitive markets or by adopting or maintaining procedures that appropriately value the objectively demonstrated therapeutic significance of a pharmaceutical.	(c) sound economic incentives and competitive markets for the efficient development of and access to patented and generic pharmaceutical products and medical devices;	(d) the need to recognise the value of pharmaceutical products and medical devices through the operation of competitive markets or by adopting or maintaining procedures that appropriately value the objectively demonstrated therapeutic significance of a pharmaceutical product or medical device.

As presented in Table 2, the agreements state four common areas to achieve these aims: public health, research and development, (timely and affordable) access, and recognizing value. First, they state the importance of pharmaceutical products (and medical devices) to promoting public health. However, some variations are noted. The AUSFTA covers only “*innovative*” pharmaceutical products, while the others include pharmaceutical products and “*medical devices*”. The KORUS includes not only patented pharmaceuticals but also generics in the agreement. Second, the agreements clarify the importance of research and development in the pharmaceutical industry with appropriate government support, such as intellectual property protection. The KORUS describes incentives for innovation other than intellectual property protection. However, the KORUS does not clarify other incentives beyond intellectual property protection. Third, the agreements underscore “timely and affordable” access. Two themes should be noted to understand access: the subject of the access and the specific methods to achieve “timely and affordable” access. The AUSFTA states that “*innovative*” pharmaceuticals are the subject of access, and it stresses transparent, accountable and “*expeditious*” procedures, while the KORUS states that “*safe and effective*” pharmaceuticals and medical devices are the subject of access, and it emphasizes transparent and accountable procedures. The TPP clarifies that pharmaceuticals and medical devices are the subject, and it stresses transparent, accountable, “*expeditious and impartial*” procedures. Finally, the agreements discuss how to recognize the value of the medicines. The AUSFTA and TPP provide two methods, either “*competitive markets*” or “*procedures that appropriately value objectively demonstrated therapeutic significance*”. However, the KORUS states one method, which is very similar to the *“competitive markets”* in the AUSFTA and TPP: sound economic incentives and competitive markets. The remaining method, which is *“procedures that appropriately value objectively demonstrated therapeutic significance”*, is stated in a separate provision of the KORUS, entitled “Access to Innovation”.

### Innovation: value for money

Innovation in medicines is closely related to drug development or regulatory processes, but it has little connection with pricing and reimbursement processes. To fill this gap, we defined innovation during the pricing and reimbursement process as higher therapeutic value for a given price, which is linked to value for money. As already explained, the agreements include “*recognizing the value*” in the domain of principles. Furthermore, the agreements cover substantial methods for determining the reimbursement decision for the newly marketed drug and setting the price of the drug in the domain of value for money.

The KORUS provides separate provisions related to innovation. Article 5.2, which is entitled “Access to innovation”, is composed of three parts. First, it clarifies that the procedures, rules, criteria, and guidelines related to pricing and reimbursement decisions should be fair, reasonable, and nondiscriminatory. This provision is similar to provisions on transparency, which applies to the procedures of pricing and reimbursement decisions. However, note that the KORUS includes rules, criteria, and guidelines, which are beyond the procedures, as the subjects of recognizing value. Second, it provides two methods for recognizing the value of medicines, based on either competitive market-derived prices or on nonmarket-derived prices.^1^ Interestingly, the latter describes the principles of health technology assessments. Thus, this clause offers clues for interpreting “*procedures that appropriately value the objectively demonstrated therapeutic significance*”, as stated in the AUSFTA and TPP. Finally, it offers procedures for applying for the reimbursement process based on additional indications for the medicine.

### Transparency

The domain of transparency applies to the procedures of pricing and reimbursement decisions. Compared to the domain of value for money, transparency is described in detail in the agreements. Interestingly, the heading of the provisions changed from “transparency” to “procedural fairness”, and the content was articulated in the TPP.

Table 3 presents comparison of transparency provisions in the AUSFTA, KORUS, and TPP. We categorized the transparency provisions into five parts: timely decision, disclosure rules, opportunity to provide comments, provision of information, and review process. First, the agreements state that requests for the listing of the medicine by a manufacturer must be completed within a specific time. The KORUS adds the word “*reasonable*” to timely decisions. Second, disclosure rules dictate that rules, methodologies, principles, and guidelines be disclosed to manufacturers and the public. The KORUS also adds “criteria (including those used, if any, to determine comparator products)” to the list of disclosure, and it is closely related to health technology assessment. Third, the agreements guarantee applicants’ participation in providing comments at relevant points during the pricing and reimbursement procedures. The KORUS provides applicants with timely and “meaningful” opportunities to provide comments, which would provide the chance for industry to exert influence on pricing and reimbursement decisions. The TPP states that the public, if appropriate, could provide comments at relevant points. Fourth, the provision of information can be divided into manufacturers and the public. The agreements state that the government should provide “*detailed*” written information to the manufacturers regarding the basis for decisions. Furthermore, the KORUS requires the government to provide “*meaningful*” information to manufacturers. In contrast, the AUSFTA and TPP request the government to provide written information to the public. Finally, the agreements state that an independent review process can be invoked at the request of the manufacturer that is directly affected by a decision made by the government. The TPP includes “*an internal review process*” in its provisions.

**Table 3:** Comparison of transparency provisions in AUSFTA, KORUS, and TPP

		AUSFTA	KORUS	TPP
Timely decision		(a) ensure that consideration of all formal proposals for listing are completed within a specified time;	(a) ensure that consideration of all formal requests for the pricing or approval of pharmaceutical products or medical devices for reimbursement is completed within a *reasonable,* specified period;	(a) ensure that consideration of all formal and duly formulated proposals for such listing of pharmaceutical products or medical devices for reimbursement is completed within a specified period of time;
Disclosure rules		(b) disclose procedural rules, methodologies, principles, and guidelines;	(b) disclose to applicants within a reasonable, specified period all procedural rules, methodologies, principles, *criteria*, and guidelines;	(b) disclose procedural rules, methodologies, principles and guidelines;
Opportunities to provide comments		(c) afford applicants timely opportunities to provide comments at relevant points;	(c) afford applicants timely and *meaningful* opportunities to provide comments at relevant points;	(c) afford applicants and, if appropriate, the public, timely opportunities to provide comments at relevant points;
Provision of information	to applicants	(d) provide applicants with detailed written information regarding the basis for recommendations or determinations;	(d) within a reasonable, specified period, provide applicants with *meaningful*, detailed written information regarding the basis for recommendations or determinations;	(d) provide applicants with written information sufficient to comprehend the basis for recommendations or determinations;
	to public	(e) provide written information to the public regarding its recommendations or determinations;	*Not Applicable*	(f) provide written information to the public regarding recommendations or determinations
Review process		(f) make available an independent review process that may be invoked at the request of an applicant directly affected by a recommendation or determination.	(e) make available an independent review process that may be invoked at the request of an applicant directly affected by a recommendation or determination;	(e) make available: (i) an independent review process; or (ii) *an internal review process*; and
Others		Side letters PBS 3 (c) expedited procedures for processing of applications not requiring an economic evaluation 4. provide opportunities to apply for an adjustment to the price of a pharmaceuticals.	(f) make all reimbursement decision-making bodies open to all stakeholders; and (g) make publicly available the membership list of all committees.	

Additionally, the AUSFTA states expedited procedures for processing applications not requiring an economic evaluation and opportunities to apply for an adjustment to the price of pharmaceuticals. Note that the latter is associated with the domain of value for money. The KORUS describes reimbursement decision-making bodies. In particular, the decision-making body is open to all stakeholders and the membership list of all committees is to be publicly available.

### Transforming international agreements into a numerical representation

In this section, we conducted a computational text analysis to supplement the aforementioned content analysis. We selected two domains in the framework, namely, principles and transparency, provided word frequencies, and applied correlation testing to quantify the similarities between the AUSFTA and the KORUS and between the KORUS and the TPP.

### Word frequencies

Table 4 provides information about word frequencies in the domain of principles and transparency. Note that the subjects of the agreements, which are “innovative pharmaceuticals” for the AUSFTA and “pharmaceutical products and medical devices” for the KORUS and TPP, are frequently observed in the agreements. Additionally, we confirmed that the corresponding content is short in the AUSFTA compared to the KORUS and TPP. As already explained, some agreements emphasize specific topics, and some words are frequently observed in the agreements. For instance, “access”, “patented”, and “generic” are frequently used in the KORUS, while “public” is frequently observed in the TPP.

**Table 4:** Word frequencies in AUSFTA, KORUS, and TPP

Principles with aims and areas						Transparency					
AUSFTA		KORUS		TPP		AUSFTA		KORUS		TPP	
Pharmaceutical	5	Medical	9	Pharmaceutical	5	Pharmaceutical(s)	9	Reimbursement	12	Party	20
Quality	3	Pharmaceutical	8	Medical	5	PBAC	6	Products	12	Proposed	11
Innovative	3	Products	7	Products	4	Provide	6	Pharmaceutical	11	Agreement	9
Health	3	Devices	7	Health	4	Listing	5	Medical	11	Regulation	8
Procedure	2	Parties	7	Quality	3	Recommendations	5	Devices	11	Procedures	8
Principles	2	Health	5	Public	3	Process	4	Pricing	9	Administrative	8
Parties	2	Development	4	Importance	3	PBS	4	Regulations	8	Respect	7
Committed	2	Care	4	Devices	3	Opportunity	4	Party	8	Provide	7
Care	2	Access	4	Research	2	Information	4	Reasonable	6	Proceedings	7
		Quality	3	Procedure	2	Australia	4	Related	5	Information	7
		Patented	3	Principles	2	Application	4	Proposed	5	Review	6
		Generic	3	Parties	2	Reimbursement	3	Respecting	4	Reasonable	6
		Transparent	2	Including	2	Procedures	3	Period	4	National	6
		Safety	2	Development	2	Healthcare	3	Matter	4	Measures	6
		Research	2	Care	2	Federal	3	Level	4	Matter	6
		Promoting	2			Determinations	3	Including	4	Reimbursement	5
		Innovation	2			Applications	3	Government	4	Provided	5
		Incentives	2					Central	4	Official	5
		Improve	2					Application	4	Health	5
		Efficacy	2							Ensure	5
		Accountable	2							Comments	5

Similarly, we counted word frequencies in the domain of transparency. The TPP comprehensively articulates transparency in the dedicated chapter and its annex. Similarly, the AUSFTA describes transparency in the Annex 2-C Pharmaceuticals and Side letters PBS. We included the related chapter, annex, and side letters for analysis. The subject of the domain of transparency, which is “procedures for listing or setting the amount (or pricing) of pharmaceuticals (products and medical devices)”, is frequently observed in the AUSFTA and the KORUS. However, these words are not ranked in the top position in the TPP. Instead, other words presented in Chapter 26, entitled “Transparency and Anticorruption”, are in the top position. Specifically, Chapter 26 states provisions on publication, administrative proceedings, review and appeal, and provision of information. Thus, words related to these provisions, including “administrative”, “proceedings”, “review”, and “information”, are ranked in higher positions.

Interestingly, we found a word, “reasonable”, only in the KORUS and TPP. For instance, “reasonable time, period, or opportunity” are frequently observed in the KORUS and TPP, while the AUSFTA uses “specified time or timely”. Note that the wording is also stated in the KORUS and TPP. For instance, the KORUS reads thus: “(a) ensure that consideration of all formal requests for the pricing or approval of pharmaceutical products or medical devices for reimbursement is completed within a reasonable, *specified period*”; and the TPP reads as follows: “(a) ensure that consideration of all formal and duly formulated proposals for such listings of pharmaceutical products or medical devices for reimbursement are completed within a *specified period of time*”, indicating that the KORUS and TPP place more obligations on member countries than the AUSFTA when a government reviews formal proposals by applicants.

### Correlation testing

We quantified similarities in word frequencies among the three agreements by applying a correlation test. Note that a correlation test allows us to quantify how similar and different the sets of word frequencies are [45]. In the domain of principles, the word frequencies are more correlated between the KORUS and TPP than between the others. For instance, the correlation between the AUSFTA and the KORUS (0.5984, *p* < 0.0001) is the lowest, the correlation between the AUSFTA and the TPP (0.6660, *p* < 0.0001) is in the middle, and the correlation between the KORUS and the TPP (0.8223, *p* < 0.0001) is the highest. However, we found a different result in the domain of transparency. Interestingly, the correlation between the AUSFTA and the TPP (0.5018, p < 0.0001) is the highest, the correlation between the KORUS and the TPP (0.4511, *p* < 0.0001) is in the middle, and the correlation between the KORUS and the AUSFTA (0.4010, *p* < 0.0001) is the lowest. Note that the highest correlation between agreements, which is between the KORUS and the TPP, is marginal when compared to the domain of principles.

## Discussion

Currently, health systems are struggling with the marketing of high-priced medicines and sudden price increases in generics [1–4]. Governments have adopted various policy options in pricing and reimbursement schemes to reduce the impact of high-priced medicines [5–8, 46], and some of these options are currently moving under the jurisdiction of international agreements [25–27]. This study addressed the trends in international agreements regarding pricing and reimbursement decisions and suggested their implications from the perspective of access to medicines. To capture the trends, we formulated a framework composed of principles, innovation (value for money), and transparency and conducted a detailed content analysis and a computational text analysis.

### Limitations and strengths of the study

This study provides several strengths in interpreting international agreements. First, our analysis has a specific interest in provisions related to pricing and reimbursement policy. Furthermore, we suggested three domains, namely, principles, innovation (value for money), and transparency, to understand provisions regarding pricing and reimbursement. Second, we introduced a computational text analysis to complement a content analysis and suggested word frequencies and correlations among the subjects to note trends in international agreements.

This study has several limitations. First, this study breaks down complex trade agreements to the specific area of provisions related to the domestic pricing and reimbursement policy for new medicines. One may argue that interpretations of the contents of the provisions need to be situated in a whole context and interpreted within the relevant part of the text. For instance, the technical barriers to trade in international agreements, which address regulations, standards, testing and certification procedures, might be relevant in the marketing authorization of new drugs. Similarly, government procurement in international agreements might overlap with the pricing and reimbursement of medicines. However, it is noteworthy that several studies have analyzed “overall” characteristics of the “CPTPP” and their potential impact on pharmaceutical policy [25–28], indicating that efforts to understand a specific part of a whole agreement are still necessary. Furthermore, health systems are struggling with unexpected drug expenditure. Thus, this study selected the pricing and reimbursement for new medicines as a specific topic. Second, this study only analyzed the trends in international agreements on pricing and reimbursement. However, considerable “constructive ambiguities” are observed in international agreements [11], which leave variations in interpretation during the implementation process at the domestic level. For instance, the AUSFTA designates “innovative pharmaceutical products” as the subject of the agreement in principle. However, the AUSFTA covers all medicines, not just innovative products, in practice. Similarly, one may argue that interpreting a treaty must be based on the in-depth intellectual engagement of scholars. However, it is noteworthy that transforming legal texts into data enables scholars to investigate international agreements in unprecedented depth and breadth.

### Trends in international agreements

Similar to the provisions for intellectual property [11, 25], the scope of international agreements regarding pricing and reimbursement decisions are broadened and strengthened. For instance, the AUSFTA covers only innovative pharmaceuticals, while the KORUS and TPP include medical devices as well as pharmaceuticals. Furthermore, the contents of recently signed international agreements are much more clear in describing the provisions. Provisions on transparency, which was stated under the heading of “Transparency and procedural fairness”, are comprehensively described in the TPP.

Over time, the domain of transparency has changed significantly more than the remaining domains. A correlation test confirmed that the similarities in word frequencies between the agreements in the domain of transparency continuously scored low. For instance, the values of the correlation between the AUSFTA and the KORUS and between the KORUS and the TPP are 0.4498 and 0.4511, respectively, indicating that the contents of the agreements have not converged over time. Note that the TPP meaningfully stresses transparency in the agreement with detailed form and the headings of the chapter and annex, entitled “Transparency and Anticorruption” and “Transparency and Procedural Fairness for Pharmaceutical Products and Medical Devices”, respectively. According to the provisions, pharmaceutical companies have more opportunities to advocate for their interests and arguments, to influence opinions during the pricing and reimbursement decision processes, to investigate the decisions on listings and setting amounts of reimbursement and to challenge decisions previously rendered. Additionally, interesting words, such as “meaningful” opportunity, “all” procedural rules, and “reasonable” time, have appeared in recently ratified agreements, favoring companies over governments.

Conversely, the correlation coefficient of word frequencies in the domain of principles increased over time. The values between the AUSFTA and the KORUS and between the KORUS and the TPP are 0.5984 and 0.8223, respectively, demonstrating that the similarities of word frequencies between the agreements in the domain of principles increased. We also confirmed that the principles of the agreements in the AUSFTA and KORUS are sustained in the TPP. The eligible agreements emphasize “facilitating high quality health care” and “improved public health of their nationals” through public health, research and development, timely and affordable access, and recognizing value. Moreover, interesting words, such as “impartial and expeditious” procedures, appear in recently ratified agreements, also favoring companies over governments.

Finally, the domain of innovation (value for money) is observed only in the KORUS. The AUSFTA and TPP state the corresponding provisions in the domain of principles or transparency. The AUSFTA and TPP acknowledge “recognizing the value through (1) the operation of competitive markets or (2) by adopting procedures that appropriately value the objectively demonstrated therapeutic significance” in the domain of principle. However, they do not state specific methods to measure the value objectively demonstrating therapeutic significance. The KORUS provides clues for interpreting this provision: health technology assessments. For instance, the KORUS states “permit a manufacturer of the pharmaceutical product or medical device to apply, based on evidence of safety or efficacy, for an increased amount of reimbursement over that provided for comparator products, if any, used to determine the amount of reimbursement;”. Furthermore, the TPP states that companies can participate and voice their interests in the review process of listing applications, as well as in establishing laws, regulations, and procedures in the domain of transparency, indicating that a manufacturer has more chances to promote its interests in introducing laws, regulations, and procedures regarding reimbursement processes.

### Characteristics of the (CP)TPP

The TPP is a plurilateral agreement among 12 countries, including the United States and low- and middle-income countries, while the AUSFTA and KORUS are bilateral agreements between high-income countries. The United States, which is the main member of the agreements, has been pursuing efforts to ratchet up standards of international agreements, including aspects such as intellectual property and other trading norms [37].

It is interesting to note trends in the investment chapter in the TPP. The investment chapter included in the TPP is not novel compared to similar chapters in other international agreements signed a decade earlier [35]. Approximately, 80% of the main text of the TPP has been copied and pasted from the investment chapter of the USA-Colombia Free Trade Agreement, indicating that the TPP implies a treaty made by the United States. The remaining 20% of the main text of the TPP is due to a range of substantive clarifications, and the only truly novel provisions are corporate social responsibilities. In our study, we found consistent results. The principles of provisions on pricing and reimbursement in the TPP are not novel compared to those in the KORUS, which was signed a decade earlier. However, the provisions related to transparency are more articulated in the TPP than in the AUSFTA and KORUS. The articulations (or clarifications) in the TPP might be the outcome of it being a plurilateral agreement, which requires extensive and protractive negotiations among member countries with competing interests.

### Implications for access to medicines

Underscoring the principles of the agreements is noteworthy for identifying the policy implications of international agreements for access to medicines. The international agreements state that “high quality health care and improved public health” are the basic principles of the agreements. Furthermore, they emphasize that timely and affordable access to new medicines is essential to achieving the aims. The three agreements state the importance of “timely and affordable access to pharmaceutical products” in the domain of principles. For instance, the TPP states “the need to promote timely and affordable access to pharmaceutical products and medical devices, through transparent, impartial, expeditious and accountable procedures”. However, we could not find any provisions regarding the affordability of new medicines in the agreements. Specifically, the word “affordability” is stated only once in all three agreements, indicating that affordability at the individual or payer level should be closely considered when negotiating agreements or adopting international agreements at the country level through domestic legislation.

The TPP includes “transparency and procedural fairness” in the agreements. Procedural fairness is concerned with the procedures used in decision making, rather than the actual outcomes reached by the decisions [47–49]. Generally, the rule of procedural fairness requires that applicants 1) be provided with a fair and unbiased assessment of their applications, 2) be informed of the decision makers’ concerns, and 3) have a meaningful opportunity to express concerns about their applications. Thus, procedural fairness, as stated in the TPP, including timely decision without undue delay, disclosure rules, opportunities to provide comments, provision of information, and a process for requesting review, is an essential part of procedural fairness in pricing and reimbursement decisions. However, we should separate the contents of international agreements into procedural fairness and substantial fairness or fairness in the actual outcomes reached by decision, and the laws and regulations adopted for reimbursement decisions, which are closely related to the actual outcomes, should be discussed from the perspective of substantial fairness rather than procedural fairness.

Procedural fairness is desirable in making accountable health policies [48, 50]. However, we should be deliberative in selecting the stakeholders who participate in decision processes. The participation of the public, as stated in the TPP, is essential to maintaining a balanced view between private interests and public interests. Furthermore, the accountability of participants who influence decisions should be noted. As already demonstrated, the TPP gives companies more opportunities to participate in pricing and reimbursement decisions, so companies have more accountability in making decisions. Thus, confidential information, including discounts or rebates on international list prices or the costs of research and development, should be disclosed if the government needs it to recognize the real value of pharmaceuticals or to guarantee the sustainability of health systems.

## Conclusion

We found that the scope of international agreements regarding pricing and reimbursement decisions is increasingly broad and strong. Notably, transparency provisions have changed significantly over time. It is realistic to assume that recently signed international agreements favor companies over governments by underscoring procedural fairness and timely access. Pharmaceutical companies will have more opportunities to advocate for their positions, to protect their interests in pricing and reimbursement decision processes, to investigate the decisions on listings and setting the amounts of reimbursement on new medicines and medical devices previously rendered and to challenge related decisions. However, access to affordable medicines is another goal towards which international agreements aim. Furthermore, substantial fairness and the accountability of companies should be discussed when negotiating agreements or adopting international agreements through domestic legislation.

## Data Availability

Not applicable.

## Footnotes

1 “Permit a manufacturer of the pharmaceutical product or medical device to apply, based on evidence of safety or efficacy, for an increased amount of reimbursement over that provided for comparator products, if any, used to determine the amount of reimbursement;”
